# Trends in Suicide Among Youth Aged 10 to 19 Years in the United States, 1975 to 2016

**DOI:** 10.1001/jamanetworkopen.2019.3886

**Published:** 2019-05-17

**Authors:** Donna A. Ruch, Arielle H. Sheftall, Paige Schlagbaum, Joseph Rausch, John V. Campo, Jeffrey A. Bridge

**Affiliations:** 1 The Research Institute at Nationwide Children’s Hospital, Columbus, Ohio; 2 Department of Pediatrics, The Ohio State University College of Medicine, Columbus; 3 West Virginia University, School of Medicine, Morgantown; 4 Department of Psychiatry and Behavioral Health, The Ohio State University College of Medicine, Columbus

## Abstract

**Importance:**

Suicide is a leading cause of death among youth aged 10 to 19 years in the United States, with rates traditionally higher in male than in female youth. Recent national mortality data suggest this gap may be narrowing, which warrants investigation.

**Objective:**

To investigate trends in suicide rates among US youth aged 10 to 19 years by age group, sex, race/ethnicity, and method of suicide.

**Design, Setting, and Participants:**

Cross-sectional study using period trend analysis of US suicide decedents aged 10 to 19 years from January 1, 1975, to December 31, 2016. Data were analyzed for periods defined by statistically significant changes in suicide rate trends. Suicide rates were calculated using population estimates.

**Main Outcomes and Measures:**

Period trends in suicide rates by sex and age group were assessed using joinpoint regression. Incidence rate ratios (IRRs) were estimated using negative binomial regression comparing male and female suicide rates within periods.

**Results:**

From 1975 to 2016, we identified 85 051 youth suicide deaths in the United States (68 085 male [80.1%] and 16 966 female [19.9%]) with a male to female IRR of 3.82 (95% CI, 3.35-4.35). Following a downward trend until 2007, suicide rates for female youth showed the largest significant percentage increase compared with male youth (12.7% vs 7.1% for individuals aged 10-14 years; 7.9% vs 3.5% for individuals aged 15-19 years). The male to female IRR decreased significantly across the study period for youth aged 10 to 14 years (3.14 [95% CI, 2.74-3.61] to 1.80 [95% CI, 1.53-2.12]) and 15 to 19 years (4.15 [95% CI, 3.79-4.54] to 3.31 [95% CI, 2.96-3.69]). Significant declining trends in the male to female IRR were found in non-Hispanic white youth aged 10 to 14 years (3.27 [95% CI, 2.68-4.00] to 2.04 [95% CI, 1.45-2.89]) and non-Hispanic youth of other races aged 15 to 19 years (4.02 [95% CI, 3.29-4.92] to 2.35 [95% CI, 2.00-2.76]). The male to female IRR for firearms increased significantly for youth aged 15 to 19 years (χ^2^ = 7.74; *P* = .02 for sex × period interaction). The male to female IRR of suicide by hanging or suffocation decreased significantly for both age groups (10-14 years: χ^2^ = 88.83; *P* < .001 for sex × period interaction and 15-19 years: χ^2^ = 82.15; *P* < .001 for sex × period interaction). No significant change was found in the male to female IRR of suicide by poisoning across the study period.

**Conclusions and Relevance:**

A significant reduction in the historically large gap in youth suicide rates between male and female individuals underscores the importance of interventions that consider unique differences by sex. Future research examining sex-specific factors associated with youth suicide is warranted.

## Key Points

**Question:**

Does the disproportionate increase in suicide rates among female youth indicate a narrowing of the historically large gap between male and female youth in suicide?

**Findings:**

This cross-sectional study of 85 051 youth suicide deaths found a significant reduction in the gap between male and female rates of suicide among youth aged 10 to 19 in the United States, with the most pronounced narrowing in younger individuals. Female suicide rates by hanging or suffocation are approaching those of male youth, and significant differences by race/ethnicity also exist.

**Meaning:**

A narrowing gap between male and female youth suicide rates underscores the importance of early suicide prevention efforts that take both sex and developmental level into consideration.

## Teaser

This cross-sectional study investigates trends in suicide rates among US youth aged 10 to 19 years by sex, age group, race/ethnicity, and method of suicide.

## Introduction

Suicide is the second leading cause of death among youth aged 10 to 19 years in the United States, with suicide rates increasing 33% between 1999 and 2014.^1,2^ Rates of suicide in the United States have historically been higher in male individuals than in female individuals across all age groups.^3,4^ However, 2 recent reports from the Centers for Disease Control and Prevention^5,6^ reveal that female youth are experiencing a greater percentage increase in suicide rates compared with male youth. A study^5^ of youth aged 15 to 19 years showed suicide rates for female individuals more than doubled from 2007 to 2015, compared with a 31% increase for male individuals; an additional report^6^ found female youth aged 10 to 14 years experienced the largest percentage increase in suicide rates compared with other age groups, tripling from 0.5 per 100 000 in 1999 to 1.5 per 100 000 in 2014. Although informative, these reports do not address the extent to which the disproportionate increase in suicide rates among female youth is contributing to a narrowing gap between male and female youth suicide rates. Understanding disparities in youth suicide rates is crucial for developing targeted prevention strategies.^7^ This study examines these trends by investigating age-specific data by sex, race/ethnicity, method of suicide, and US regions using the most recent national mortality data available through 2016.

## Methods

Deidentified data were obtained from Wide-ranging Online Data for Epidemiologic Research (WONDER) records in which suicide (coded as E950-E959 for the eighth and ninth revisions of the *International Classification of Diseases *[1975-1998] and X60-X84, Y87.0, and U03 for the *International Classification of Diseases, Tenth Revision* [1999-2016]) was listed as the underlying cause of death among youth aged 10 to 19 years.^8^ The age group and period were selected to facilitate comparison with previous analyses^3,4,5^ and provide sufficient context for evaluating trends in suicide rates. Crude rates per 100 000 persons were calculated using WONDER population estimates. This article follows the Strengthening the Reporting of Observational Studies in Epidemiology (STROBE) guideline for cross-sectional studies.^9^ The study was not considered human research according to the review policy of the Research Institute at Nationwide Children’s Hospital institutional review board because all data were deidentified and publicly available.

Trends in suicide rates by sex and age group (10-14 years and 15-19 years) were assessed using Joinpoint regression software version 4.3.1.0 (Surveillance Research Program, National Cancer Institute). Incidence rate ratios (IRRs) and corresponding 95% confidence intervals were estimated using negative binomial regression comparing suicide rates between male and female individuals within select periods. Comparisons of the male to female IRR for each period were performed using the χ^2^ test to identify statistically significant trends among demographic subgroups. Statistical analyses were conducted using Stata/IC statistical software version 14.1 (StataCorp Inc) and a 2-tailed significance level of *P* < .05.

## Results

**Figure 1.**
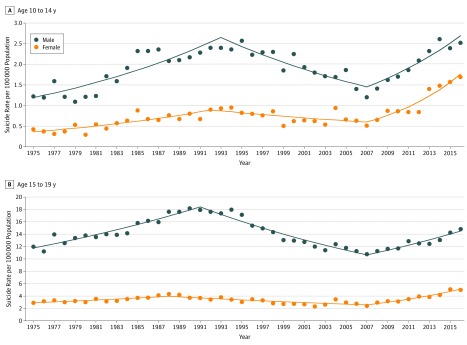
Suicide Trends Among Youth Aged 10 to 19 Years in the United States, 1975 to 2016 Suicide rate trends are displayed as linear segments connected at the joinpoint or year when the slope of each trend changes significantly. Data markers indicate observed rates and solid colored lines indicate model rates.

Between 1975 and 2016, a total of 85 051 suicide deaths were identified for youth aged 10 to 19 years in the United States (68 085 male [80.1%] and 16 966 female [19.9%]), with a male to female IRR of 3.82 (95% CI, 3.35-4.35). Beginning in 1975, the trend in suicide rates for youth aged 10 to 14 years increased 5.4% annually for female individuals until 1992, and 4.5% annually for male individuals until 1993 (Table and Figure 1A). Following declining trends for both sexes until 2007, the trend in suicide rates for female youth showed the largest significant percentage change, increasing 12.7% annually compared with 7.1% for male youth, contributing to a discernable narrowing of the gap between male and female rates. The decrease in the male to female IRR for suicide among youth aged 10 to 14 years from 3.14 (95% CI, 2.74-3.61) in 1975 to 1991 to 1.80 (95% CI, 1.53-2.12) in 2007 to 2016 was statistically significant (χ^2^ = 30.41; *P* < .001 for sex × period interaction) (eTable 1 in the Supplement). The male to female IRR of suicide declined across all US regions over time, although not significantly in the West (eTable 1 in the Supplement).

**Table.** Trends in Suicide Rates (Per 100 100) Among Youth Aged 10 to 19 Years in the United States, 1975 to 2016^a^

Age	Sex	Segment 1	Annual Change, % (95% CI)	Segment 2	Annual Change, % (95% CI)	Segment 3	Annual Change, % (95% CI)
10-14 y	Female	1975-1992	5.42 (3.25 to 7.63)	1992-2007	−2.65 (−4.74 to −0.51)	2007-2016	12.73 (8.81 to 16.80)
	Male	1975-1993	4.54 (3.42 to 5.68)	1993-2007	−4.21 (−5.69 to −2.70)	2007-2016	7.08 (4.21 to 10.03)
15-19 y	Female	1975-1988	2.37 (1.13 to 3.62)	1988-2007	−2.22 (−2.98 to −1.46)	2007-2016	7.91 (5.84 to 10.02)
	Male	1975-1991	2.84 (2.34 to 3.34)	1991-2007	−3.36 (−3.89 to −2.83)	2007-2016	3.49 (2.28 to 4.72)
All	Female	1975-1988	2.78 (1.48 to 4.10)	1988-2007	−2.17 (−2.94 to −1.40)	2007-2016	8.93 (6.87 to 11.04)
	Male	1975-1990	3.10 (2.55 to 3.66)	1990-2007	−3.11 (−3.58 to −2.64)	2007-2016	3.83 (2.63 to 5.04)

^a^ Suicide rate trends by age group were determined using joinpoint regression. The number and year of joinpoints associated with trends are defined statistically, and the periods for each linear segment may vary. The annual percentage change describes the rate of change for each linear segment trend.

Among youth aged 15 to 19 years, the trend in suicide rates from 1975 increased 2.4% annually for female individuals until 1988, and 2.8% for male individuals until 1991, then decreased 2.2% for female individuals and 3.4% for male individuals each year until 2007 (Table and Figure 1B). From 2007 to 2016, both sexes experienced an upward trend, increasing 7.9% per year for female youth and 3.5% for male youth. Differences in the male to female IRR across the study period were significant (χ^2^ = 20.89; *P* < .001 for sex × period interaction), increasing from 4.15 (95% CI, 3.79-4.54) in 1975 to 1987 to 4.56 (95% CI, 4.18-4.97) in 1988 to 2006, then decreasing to 3.31 (95% CI, 2.96-3.69) through 2016 (eTable 1 in the Supplement). A significant decreasing trend was found across all US regions.

**Figure 2.**
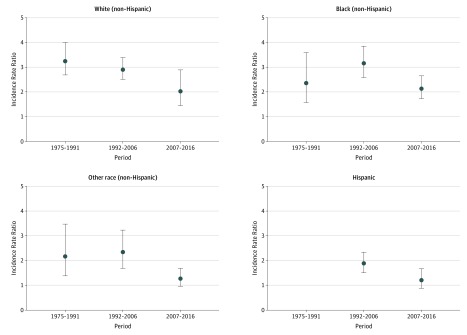
Male to Female Incidence Rate Ratios of Suicide Rates Among Youth Aged 10 to 14 Years in the United States, 1975 to 2016, by Race/Ethnicity Circles indicate the estimated natural logarithm of the period-specific incidence rate ratio and vertical lines, the 95% confidence intervals. The reference group is female youth. The 95% confidence intervals that do not include 0 are considered statistically significant. Periods reflect significant linear segment trends associated with female youth in joinpoint analyses. Other race includes American Indian or Alaskan Native and Asian or Pacific Islander. Information on Hispanic ethnicity was only available for 1990 onward and excludes data from the following states and years: Alabama, 1990; Oklahoma, 1990-1996; New Hampshire, 1990-1992; and Louisiana, 1990-1991.

When examined by race/ethnicity, the male to female IRR of suicide among youth aged 10 to 14 years declined significantly for non-Hispanic white youth (χ^2^ = 8.10; *P* = .02 for sex × period interaction) and non-Hispanic youth of other races (χ^2^ = 8.79; *P* = .02 for sex × period interaction) (Figure 2; eTable 1 and eTable 2 in the Supplement). Non-Hispanic white youth experienced the most consistent declining trend with the male to female IRR of suicide decreasing from 3.27 (95% CI, 2.68-4.00) in 1975 to 1991 to 2.04 (95% CI, 1.45-2.89) in 2007 to 2016. There were no significant differences in the male to female IRR of suicide for younger non-Hispanic black or Hispanic youth.

**Figure 3.**
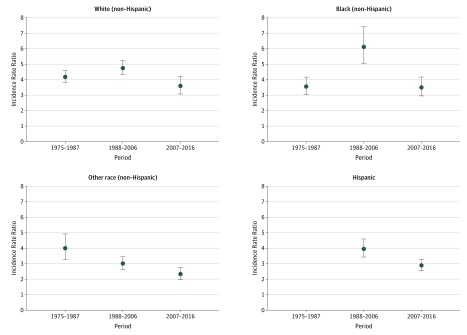
Male to Female Incidence Rate Ratios of Suicide Rates Among Youth Aged 15 to 19 Years in the United States, 1975 to 2016, by Race/Ethnicity Circles indicate the estimated natural logarithm of the period-specific incidence rate ratio and vertical lines, 95% confidence intervals. The reference group is female youth. The 95% confidence intervals that do not include 0 are considered statistically significant. Periods reflect significant linear segment trends associated with female youth in joinpoint analyses. Other race includes American Indian or Alaskan Native and Asian or Pacific Islander. Information on Hispanic ethnicity was only available for 1990 onward and excludes data from the following states and years: Alabama, 1990; Oklahoma, 1990-1996; New Hampshire, 1990-1992; and Louisiana, 1990-1991.

Analyses among youth aged 15 to 19 years showed significant changes in the male to female IRR of suicide for non-Hispanic white youth (χ^2^ = 11.60; *P* = .003 for sex × period interaction), non-Hispanic black youth (χ^2^ = 24.66; *P* < .001 for sex × period interaction), and non-Hispanic youth of other races (χ^2^ = 16.81; *P* < .001 for sex × period interaction) (Figure 3; eTable 1 and eTable 3 in the Supplement). Differences in the male to female IRR were greatest for non-Hispanic black youth, with an increasing trend between 1975 to 1987 and 1998 to 2006, followed by a declining trend in the male to female IRR from 2007 to 2016. The male to female IRR of suicide decreased significantly for non-Hispanic youth of other race from 4.02 (95% CI, 3.29-4.92) in 1975 to 1987 to 2.35 (95% 5 CI, 2.00-2.76) in 2007 to 2016. A significant downward trend in the male to female IRR was also observed for Hispanic youth aged 15 to 19 years (χ^2^ = 8.75; *P* = .003 for sex × period interaction), but data were only available for the 1998 to 2006 and 2007 to 2016 reporting periods.

**Figure 4.**
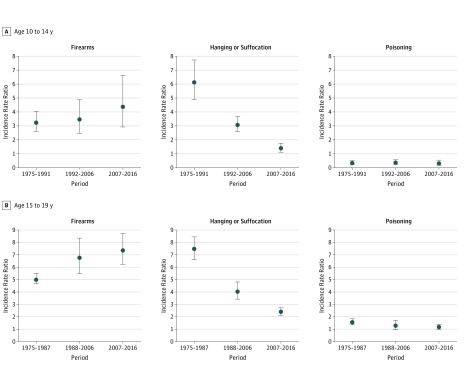
Male to Female Incidence Rate Ratios of Suicide Rates Among Youth Aged 10 to 19 Years in the United States, 1975 to 2016, by Method Circles indicate the estimated natural logarithm of the period-specific incidence rate ratio and vertical lines, 95% confidence intervals. The reference group is female youth. The 95% confidence intervals that do not include 0 are considered statistically significant. Periods reflect significant linear segment trends associated with female youth in joinpoint analyses.

When examining method of suicide, the male to female IRR for firearms increased significantly for youth aged 15 to 19 years (χ^2^ = 7.74; *P* = .02 for sex × period interaction) (Figure 4B; eTable 1 in the Supplement). The male to female IRR of suicide by hanging or suffocation decreased significantly for both age groups (10-14 years: χ^2^ = 88.83; *P* < .001 for sex × period interaction and 15-19 years: χ^2^ = 82.15; *P* < .001 for sex × period interaction). No significant change was found in the male to female IRR of suicide by poisoning across the study period. Changes in the male to female IRR of suicide by other methods was significant for youth aged 15 to 19 years (χ^2^ = 9.96; *P* = .007 for sex × period interaction).

## Discussion

The core finding of this study is that the ratio of male to female suicide rates for children and adolescents has declined over the past 40 years. These results expand on previous reports of a disproportionate increase in the suicide rate among female relative to male youth^5,6^ and highlight a significant reduction in the historically large gap in suicide rates between sexes. Following a downward trend in suicide rates for both sexes in the early 1990s, increasing rates of youth suicide since 2007 have been associated with an accelerated narrowing of the gap between male and female rates, with the largest percentage increase in younger female individuals. These trends were observed across all regions in the United States.

Consistent with earlier studies,^9^ our findings provide evidence of racial/ethnic disparities in youth suicide rates among male and female individuals. The male to female IRR of suicide decreased for all racial/ethnic categories since 2007, with a significant declining trend across the study period in younger non-Hispanic white youth and older non-Hispanic youth of other races. Future research to identify sex-specific risk factors for youth suicide and distinct mechanisms of suicide in male and female individuals within racial/ethnic groups could lead to improved suicide prevention strategies and interventions.

A particularly important finding relates to changes in method of suicide, with hanging or suffocation showing a greater increase as the cause of death among female relative to male youth. Consistent with previous reports of increasing rates of suicide by hanging or suffocation in female youth,^3,10,11^ the male to female IRR of suicide by hanging or suffocation declined significantly for both age groups. It is troubling that a growing proportion of female youth are choosing this more violent and lethal method, as it is well documented female individuals have higher rates of attempted suicide.^12,13^ Most youth suicide decedents actually die on their first attempt, with the likelihood of death on first attempt being associated with lethality of method.^14^ Consequently, a sustained shift toward a highly lethal method such as hanging or suffocation by female youth could have grave public health implications and drive elevations in the rates of female suicide. The increasing trend in differences between male and female suicide rates by firearms across the study period highlights the continuing need for prevention strategies aimed at restricting access to lethal means. The importance of poisoning as a means of suicide in female youth has been well established,^4,6,10^ and no significant changes were observed in the proportion of female individuals dying by self-poisoning across the study period.

The narrowing gap between male and female rates of suicide was most pronounced among youth aged 10 to 14 years, underscoring the importance of early prevention efforts that take both sex and developmental level into consideration. Results from this study potentially challenge the existing paradox of suicidal behavior where female individuals have higher rates of suicidal ideation and attempted suicide than male individuals, while death by suicide is lower in female individuals than male individuals.^15^ This may be especially true within some demographic groups.

Differential increases over time in risk factors for suicide among female compared with male youth could contribute to the observed increase in female suicide rates. A history of suicidal behavior is a leading predictor of future suicide in youth,^16,17^ and although rates of hospitalization for suicidal ideation and suicide attempts in youth have increased over time in both sexes, this increase has been greatest among female youth.^18^ Similarly, trends from the 2007 to 2017 national Youth Risk Behavior Survey^19^ revealed a significantly larger percentage increase in female youth who seriously considered attempting suicide (18.7% to 22.1%) compared with male youth (10.3% to 11.9%). The percentage of female youth who made a suicide plan also increased significantly from 13.4% to 17.1%, while no significant change was found in male youth.^19^ Research^20,21,22^ has also identified a strong link between youth suicide and mental health, most commonly depression. The Youth Risk Behavior Survey^19^ found that the percentage of female youth who experienced persistent feelings of sadness or hopelessness increased significantly between 2007 and 2017 (from 35.8% to 41.1%), with no significant changes seen in male youth. In addition, our results that show an increase in female suicide rates by hanging or suffocation support evidence linking direct access or proximity to more lethal means with increased rates of suicide.^23^

### Limitations

This study is not without limitations. First, these data cannot explain underlying reasons for the narrowing of the gap between the sexes in youth suicide. Second, variations in cause of death coding between the *International Classification of Diseases* eighth, ninth, and tenth revision coding systems used in this study may have affected results, despite high levels of agreement across these revisions.^24,25^ Third, it is unknown whether the accuracy of classification of a death as suicide in youth has changed during the study period. Fourth, early suicide trends for Hispanic and non-Hispanic categories should be interpreted with caution, as these data were not captured before 1990 and excluded from select states through 1996.^7^

## Conclusions

The findings of this study reveal a significant and disproportionate increase in suicide rates for female youth relative to male youth, particularly in younger individuals. Rates of suicide by hanging or suffocation and in some racial/ethnic groups among female youth are now approaching those of male youth. This narrowing gap underscores the urgency to identify suicide prevention strategies that address the unique developmental needs of female youth. Future research is warranted to examine sex-specific risk and protective factors associated with youth suicide and how these determinants can inform interventions.

## Notes

Supplement. **eTable 1.** Incidence Rate Ratios (IRR) of Male to Female Suicide Rates Among Youth Aged 10-19 Years in the United States, 1975-2016 **eTable 2.** Trends in Suicide Rates (per 100,000) Among Youth Aged 10 to 14 Years in the United States, 1975-2016 **eTable 3.** Trends in Suicide Rates (per 100,000) Among Youth Aged 15 to 19 Years in the United States, 1975-2016
