# Error in Supplement

**DOI:** 10.1001/jamanetworkopen.2019.7687

**Published:** 2019-06-21

In the Original Investigation titled “Trends in Suicide Among Youth Aged 10 to 19 Years in the United States, 1975 to 2016,”^1^ published May 17, 2019, extra rows of data were included for the numbers and rates for the South and West regions in eTable 2 in the Supplement. This article has been corrected.^1^
