# Supplementary material for: Trends in Suicide Among Youth Aged 10 to 19 Years in the United States, 1975 to 2016
Source: JAMA Netw Open. 2019 May 17;2(5):e193886. doi: 10.1001/jamanetworkopen.2019.3886 (PMC6537827; doi:10.1001/jamanetworkopen.2019.3886)
Supplement: Supplement. — eTable 1. Incidence Rate Ratios (IRR) of Male to Female Suicide Rates Among Youth Aged 10-19 Years in the United States, 1975-2016 eTable 2. Trends in Suicide Rates (per 100,000) Among Youth Aged 10 to 14 Years in the United States, 1975-2016 eTable 3. Trends in Suicide Rates (per 100,000) Among Youth Aged 15 to 19 Years in the United States, 1975-2016 [file jamanetwopen-2-e193886-s001.pdf]

Supplementary Online Content

Ruch DA, Sheftall AH, Schlagbaum P, Rausch J, Campo JV, Bridge JA. Trends in suicide among youth aged 10 to 19 years in the United States, 1975 to 2016. *JAMA Netw Open*. 2019;2(5):e193886. doi:10.1001/jamanetworkopen.2019.3886

eTable 1. Incidence Rate Ratios (IRR) of Male to Female Suicide Rates Among Youth Aged 10-19 Years in the United States, 1975-2016
eTable 2. Trends in Suicide Rates (per 100,000) Among Youth Aged 10 to 14 Years in the United States, 1975-2016
eTable 3. Trends in Suicide Rates (per 100,000) Among Youth Aged 15 to 19 Years in the United States, 1975-2016

This supplementary material has been provided by the authors to give readers additional information about their work.

© 2019 Ruch DA et al. *JAMA Network Open.*

eTable 1. Incidence Rate Ratios (IRR) of Male to Female Suicide Rates Among Youth Aged 10-19 Years in the United States, 1975-2016 [a]

	IRR (95% CI)		IRR (95% CI)		IRR (95% CI)		Sex by Time Period Interaction	
	1975 - 1991		1992 - 2006		2007 - 2016		χ^2	*P*
**Ages 10-14**	3.14	(2.74 - 3.61)	2.74	(2.45 - 3.07)	1.80	(1.53 - 2.12)	30.41	<.001
Race / Ethnicity [b]								
White	3.27	(2.68 - 4.01)	2.92	(2.52 - 3.38)	2.04	(1.45 - 2.89)	8.10	.017
Black	2.37	(1.57 - 3.59)	3.16	(2.59 - 3.85)	2.14	(1.73 - 2.65)	4.17	.125
Other [c]	2.19	(1.38 - 3.48)	2.35	(1.70 - 3.24)	1.29	(0.98 - 1.70)	8.79	.012
Hispanic [d]	1.82	(0.72 - 4.55)	1.88	(1.50 - 2.36)	1.21	(0.88 - 1.67)	5.39	.068
Method								
Firearm	3.24	(2.60 - 4.04)	3.48	(2.47 - 4.89)	4.40	(2.92 - 6.63)	1.72	.424
Hanging/Suffocation	6.17	(4.92 - 7.74)	3.09	(2.60 - 3.67)	1.42	(1.13 - 1.78)	88.83	<.001
Poisoning	0.34	(0.22 - 0.50)	0.36	(0.23 - 0.58)	0.30	(0.17 - 0.54)	0.25	.882
Other [e]	1.23	(0.79 - 1.91)	1.20	(0.82 - 1.75)	1.39	(0.71 - 2.71)	0.18	.914
Region								
Northeast	4.41	(3.36 - 5.78)	2.99	(2.40 - 3.73)	1.38	(1.02 - 1.87)	36.53	<.001
Midwest	3.60	(2.80 - 4.62)	2.84	(2.46 - 3.27)	1.81	(1.42 - 2.32)	17.76	<.001
South	2.69	(2.20 - 3.29)	3.15	(2.67 - 3.70)	1.97	(1.42 - 2.74)	8.10	.012
West	2.81	(2.20 - 3.58)	2.27	(1.88 - 2.75)	1.96	(1.51 - 2.54)	4.50	.105
	1975 - 1987		1988 - 2006		2007 - 2016		χ^2	*P*
**Ages 15-19**	4.15	(3.79 - 4.54)	4.56	(4.18 - 4.97)	3.31	(2.96 - 3.69)	20.89	<.001
Race / Ethnicity [b]								
White	4.18	(3.81 - 4.58)	4.75	(4.32 - 5.22)	3.59	(3.07 - 4.21)	11.60	.003
Black	3.58	(3.07 - 4.18)	6.13	(5.07 - 7.42)	3.51	(2.95 - 4.18)	24.66	<.001
Other [c]	4.02	(3.29 - 4.92)	3.01	(2.62 - 3.45)	2.35	(2.00 - 2.76)	16.81	<.001
Hispanic [d]	-		3.97	(3.43 - 4.59)	2.89	(2.54 - 3.28)	8.75	.003
Method								
Firearm	5.09	(4.69 - 5.51)	6.77	(5.48 - 8.37)	7.39	(6.26 - 8.73)	7.74	.021
Hanging/Suffocation	7.47	(6.60 - 8.45)	4.07	(3.43 - 4.82)	2.41	(2.13 - 2.74)	82.15	<.001
Poisoning	1.58	(1.35 - 1.85)	1.29	(0.97 - 1.73)	1.16	(0.98 - 1.37)	2.55	.279
Other [e]	2.45	(2.15 - 2.79)	3.40	(3.01 - 3.84)	2.65	(2.06 - 3.41)	9.96	.007
Region								
Northeast	4.94	(4.46 - 5.46)	4.72	(4.16 - 5.37)	3.00	(2.61 - 3.44)	29.76	<.001
Midwest	4.65	(4.11 - 5.27)	4.78	(4.20 - 5.45)	3.45	(3.02 - 3.96)	11.80	.003
South	3.75	(3.45 - 4.08)	4.79	(4.25 - 5.39)	3.69	(3.13 - 4.35)	12.23	.002
West	3.71	(3.38 - 4.07)	4.13	(3.63 - 4.70)	3.07	(2.64 - 3.57)	9.67	.008

[a] Male-to-female incidence rate ratio (IRR) time periods reflect significant linear segment trends associated with female youth in joinpoint analyses. Comparisons of the male-to-female IRR for each time period were performed using the χ^2 test to identify significant trends among demographic subgroups.
[b] Racial groups for White, Black, and Other are non-Hispanic
[c] Includes American Indian/Alaskan Native and Asian/Pacific Islander
[d] Hispanic information only available for 1990 forward and excludes data from the following states/years: Alabama 1990; Oklahoma 1990-96; New Hampshire 1990-92; Louisiana, 1990-91
[e] Other suicide methods include fall, transportation-related, drowning, cut/pierce, fire/burn, and unspecified methods.

© 2019 Ruch DA et al. *JAMA Network Open.*

eTable 2. Trends in Suicide Rates (per 100,000) Among Youth Aged 10 to 14 Years in the United States, 1975-2016

	1975-1991				1992-2006				2007-2016			
	Male		Female		Male		Female		Male		Female	
**Ages 10-14**	**No.**	**Rate**	**No.**	**Rate**	**No.**	**Rate**	**No.**	**Rate**	**No.**	**Rate**	**No.**	**Rate**
Race / Ethnicity												
White	2350	1.85	680	0.57	2266	2.27	732	0.78	1406	2.36	651	1.15
Black	229	1.02	97	0.43	417	1.75	128	0.56	292	1.83	132	0.86
Other	78	1.61	36	0.74	162	1.96	62	0.84	121	1.82	89	1.41
Hispanic[a]	25	1.19	14	0.65	337	1.37	168	0.73	262	1.11	210	0.92
Method									2081		1082	
Firearm	1472	0.94	437	0.29	1443	0.93	394	0.27	864	0.82	188	0.19
Hanging/Suffocation	1100	0.70	170	0.11	1614	1.04	498	0.34	1146	1.08	773	0.76
Poisoning	65	0.04	185	0.12	57	0.04	144	0.10	29	0.03	92	0.09
Other[b]	45	0.03	35	0.02	68	0.04	54	0.04	42	0.04	29	0.03
Region												
Northeast	420	1.31	92	0.30	396	1.41	126	0.47	226	1.28	156	0.92
Midwest	672	1.66	179	0.46	809	2.24	271	0.79	524	2.28	276	1.26
South	947	1.77	337	0.66	1181	2.16	359	0.69	779	1.96	380	1.00
West	643	2.13	219	0.76	796	2.21	334	0.98	552	2.18	270	1.12
South	6089	13.42	1563	3.59	10225	15.21	2034	3.19	5127	12.55	1322	3.40
West	4156	16.53	1062	4.45	6883	16.37	1563	3.97	3538	13.44	1088	4.38

[a] Hispanic information only available for 1990 forward and excludes data from the following states/years: Alabama 1990; Oklahoma 1990-96; New Hampshire 1990-92; Louisiana, 1990-91.
[b] Includes American Indian/Alaskan Native and Asian/Pacific Islander

© 2019 Ruch DA et al. *JAMA Network Open.*

eTable 3. Trends in Suicide Rates (per 100,000) Among Youth Aged 15 to 19 Years in the United States, 1975-2016

	1975-1987				1988-2006				2007-2016			
	Male		Female		Male		Female		Male		Female	
**Ages 15-19**	**No.**	**Rate**	**No.**	**Rate**	**No.**	**Rate**	**No.**	**Rate**	**No.**	**Rate**	**No.**	**Rate**
Race / Ethnicity												
White	16703	14.97	3838	3.58	20918	16.78	4251	3.54	9756	15.35	2564	4.27
Black	1230	6.67	351	1.87	2945	10.61	478	1.73	1295	7.52	356	2.14
Other	556	15.98	130	3.99	1266	13.79	410	4.58	850	12.56	352	5.34
Hispanic[a]	-	-	-	-	2686	10.13	605	2.55	1935	8.29	632	2.88
Method												
Firearm	11785	8.80	2245	1.74	17614	9.32	2455	1.37	6701	6.05	861	0.82
Hanging/Suffocation	3806	2.84	493	0.38	7137	3.78	1681	0.94	5499	4.97	2164	2.06
Poisoning	2035	1.52	1241	0.96	1669	0.88	1220	0.68	638	0.58	522	0.50
Other[b]	863	0.64	340	0.26	1395	0.74	388	0.22	998	0.90	357	0.34
Region												
Northeast	3056	10.70	608	2.17	3582	10.42	729	2.21	1667	8.58	532	2.86
Midwest	5188	14.90	1086	3.21	7125	15.93	1418	3.34	3504	14.53	962	4.20
South	6089	13.42	1563	3.59	10225	15.21	2034	3.19	5127	12.55	1322	3.40
West	4156	16.53	1062	4.45	6883	16.37	1563	3.97	3538	13.44	1088	4.38

[a] Hispanic information only available for 1990 forward and excludes data from the following states/years: Alabama 1990; Oklahoma 1990-96; New Hampshire 1990-92; Louisiana, 1990-91.
[b] Includes American Indian/Alaskan Native and Asian/Pacific Islander.

© 2019 Ruch DA et al. *JAMA Network Open.*
